# Vpu Protein: The Viroporin Encoded by HIV-1

**DOI:** 10.3390/v7082824

**Published:** 2015-08-04

**Authors:** María Eugenia González

**Affiliations:** Unidad de Expresión Viral, Centro Nacional de Microbiología, Instituto de Salud Carlos III, Carretera de Majadahonda-Pozuelo Km 2, Majadahonda, Madrid 28220, Spain; E-Mail: megonzalez@isciii.es; Tel.: +34-91-822-3698; Fax: +34-91-509-7966

**Keywords:** Vpu, HIV-1, Viroporin, protein trafficking, membrane permeability, ion transport, membrane pore, antiretroviral target

## Abstract

Viral protein U (Vpu) is a lentiviral viroporin encoded by human immunodeficiency virus type 1 (HIV-1) and some simian immunodeficiency virus (SIV) strains. This small protein of 81 amino acids contains a single transmembrane domain that allows for supramolecular organization via homoligomerization or interaction with other proteins. The topology and trafficking of Vpu through subcellular compartments result in pleiotropic effects in host cells. Notwithstanding the high variability of its amino acid sequence, the functionality of Vpu is well conserved in pandemic virus isolates. This review outlines our current knowledge on the interactions of Vpu with the host cell. The regulation of cellular physiology by Vpu and the validity of this viroporin as a therapeutic target are also discussed.

## 1. Introduction

The human immunodeficiency virus type 1 (HIV-1) Viral protein U (Vpu) protein is an 81-amino acid type I transmembrane protein [1,2]. Its characteristic sequence elements together with its functioning during the virus life cycle are typical for proteins belonging to the viroporin family [3,4]. Specifically, the presence of a single transmembrane domain, a luminal amino terminus, and a cytosolic carboxy terminus in the protein architecture classifies Vpu as a member of class IA viroporins [5]. The Vpu protein is translated from a Rev-regulated bicistronic mRNA that also encodes the *env* gene. Thus, during the virus replication cycle, Vpu and envelope precursor protein (Env) proteins are produced late and in a coordinated manner [6]. The translated Vpu protein modifies the distribution and/or concentration of membrane proteins in host cells. This interaction with the host cell may account for two of the main functions of Vpu: the downregulation of the CD4 receptor from the cell surface and the enhancement of the release of progeny virions from infected cells [7,8]. This review provides an updated overview about the interaction of Vpu protein with host cell membranes and its consequences for the functional integrity of subcellular compartments. How these interactions may impact on virus spread from the host cells and also the potential of Vpu viroporin as an antiviral target are discussed.

## 2. The Role of Vpu Protein in the Spread of HIV-1 from Infected Cells

**Figure 1:**
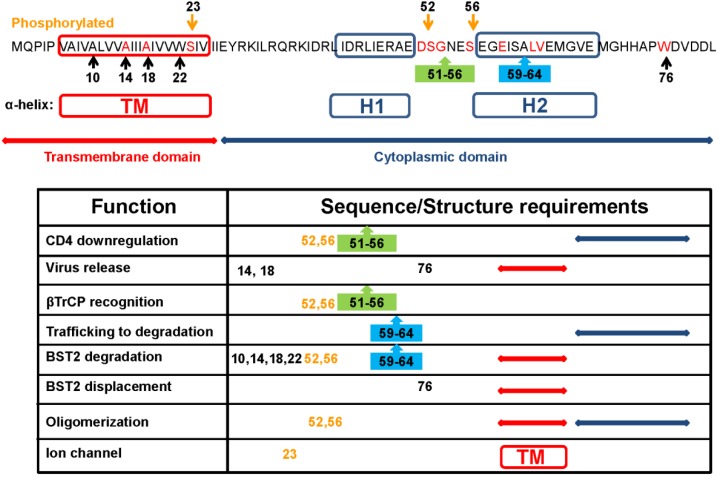
Sequence and structure requirements for Viral protein U (Vpu) functionality. Vpu from the HXB2 strain is the reference amino acid sequence showing conserved amino acid (red), motifs (boxes), structural domains, and predicted helices. Numbers refer to amino acid positions. Table summarizes the consensus published data of each function. Each function was separately characterized, using different approaches. Further coordinated studies are needed to compare requirements of these functions.

Functional analysis of full-length and truncated forms of HIV-1 Vpu pointed to a direct correlation between structure, topology, and function for this membrane protein [9]. Early studies mapped the two biological activities of Vpu protein in two separable structural domains: the transmembrane N-terminus and the cytoplasmic C-terminus [10]. Further studies showed that conserved motifs in both regions preserve the topology and supramolecular organization required for both activities (Figure 1).

### 2.1. Downregulation of CD4

HIV-1 Negative Regulatory Factor (Nef) and Vpu proteins downmodulate the CD4 receptor in infected cells. While Nef degrades pre-existing CD4 at the plasma membrane, Vpu targets the newly synthesized CD4 molecules in the endoplasmic reticulum (ER) [7]. It is well established that this modulation is independent of Env expression [11,12]. The cytoplasmic domain of the Vpu protein regulates this activity. This hydrophilic region consists of two amphipathic alpha-helices joined by a hinge region formed by the conserved DSGNES motif which can be phosphorylated by casein kinase II [13]. The phosphorylation of serines S52 and S56 is essential for this activity [14,15]. The proposed model for Vpu-induced downmodulation of cell surface CD4 is discussed below. Of note, the viral-induced downmodulation of CD4 contributes to diminish CD4-Env complexes carrying Env epitopes targeted by antibodies that mediate the antibody-dependent cell-mediated cytotoxicity (ADCC) at the cell surface [16,17]. This protection from ADCC prevents the elimination of infected cells and consequently contributes to virus propagation.

### 2.2. Enhancement of Virus Release

Vpu-induced enhancement of the release of viral particles from infected cells was first mapped to the transmembrane region of Vpu [10]. Feasible pathways explaining the promotion of virus release by Vpu are still debated. On one hand, early studies showed cell-type dependence for the promotion of productive infections by Vpu [18,19,20]. Accordingly, it was suggested that Vpu does not interact directly with viral proteins, but rather mediates this function via cellular factors. On the other hand, some other studies focused on the ion channel activity of Vpu. Thus, it was suggested that the Vpu-induced movement of monovalent cations across the internal cellular membranes creates cellular conditions, including the reorganization of raft structures, that promote budding from the plasma membrane of infected cells [21,22].

#### 2.2.1. Vpu Antagonizes BST2

The model based on antiviral host restriction has been largely developed through the identification of the interferon (IFN)-α-induced glycoprotein Bone Marrow Stromal Cell Antigen 2 (BST2) (tetherin/CD317), which inhibits the release of virus and is antagonized by Vpu [23,24]. BST2 is a type II transmembrane protein with two membrane anchors, a transmembrane domain near the N-terminus and a glycophosphatidylinositol (GPI)-linked anchor at the C terminus; both anchors are joined by an ectodomain [25]. It is thought that BST2 directly tethers viral particles to the cellular membrane [26]. Vpu removes this host restriction factor from the plasma membrane of infected cells and therefore excludes it from assembling virions [27]. The interactions between the transmembrane domains of Vpu and BST2 are proposed to be necessary for this process, leading to the degradation of BST2 in lysosomes or the proteasome [28,29]. Further, the primary sequence of the Vpu transmembrane domain governs the species-specific exclusion of BST2 [30].

Additionally, the cytoplasmic domain of Vpu is involved in the subcellular redistribution of BST2, displacing it just out of viral assembly sites at the plasma membrane [31,32]. BST2 resides in lipid rafts at the cell surface and membranes of the trans-Golgi network (TGN) [25]. Interactions between BST2 and Vpu at these cellular compartments are discussed below.

#### 2.2.2. Vpu Induces Changes in Ionic Currents of Cellular Membranes

The ion channel model was first proposed from the observation that Vpu may form homo-oligomers via the conserved hydrophobic membrane spanning helix at the transmembrane domain [33]. Moreover, structural similarities between Vpu and a second viroporin, influenza virus M2, bolstered the search for ion channel activity of this lentiviral protein in lipid bilayers [3,21,34]. Thus, computational simulation studies with the Vpu transmembrane peptide suggested that the most likely channel assembly is a pentamer [35]. Further studies in *Xenopus* oocytes revealed that Vpu forms channels that are weakly selective for monovalent cations over anions [36]. In this cellular model, mutated Vpu showed a correlation between K+ conductance alteration and CD4 degradation [37]. Nuclear magnetic resonance (NMR) spectroscopy studies revealed that the C-terminal helices modulate or promote the oligomerization of Vpu in the membrane and stabilize the conductive state of the channel [38]. Additional studies using permissive and restrictive human cells correlated Vpu-induced enhancement of viral release with membrane potential depolarization by Vpu [39]. Consequently, it is possible that the size of background K+ conductance could determine whether a particular cell type is permissive or restrictive for the infection by Vpu-defective virus. Curiously, an interaction between Vpu and the cellular weak inward K+ rectifier TASK1 was observed in cultured cells and AIDS lymphoid tissues [40]. The suppression of TASK1 conductance together with the reduction of Vpu-induced virus release from cells correlates with degradation of TASK1. Evidence that Vpu may disturb K+ transport was found also in *Saccharomyces cerevisiae* [41]. Inducible expression of Vpu impaired the growth of wild type yeast but, conversely, rescued the growth phenotype of defective K+-uptake mutants. These Vpu-promoted effects on yeast were dependent on high extracellular K+ concentration, thus, concurring with the membrane depolarization of infected cells during Vpu-enhanced virus release.

Both of the main functions of Vpu, downmodulation of CD4 and enhancement of virus release, contribute to the cell-to-cell and cell-free spread of virus [42,43]. The variable supramolecular organization and/or the turnover of Vpu may modulate distinct interactions of the viroporin with cellular membranes [44,45].

## 3. Modification of Protein Trafficking and Membrane Integrity

**Figure 2:**
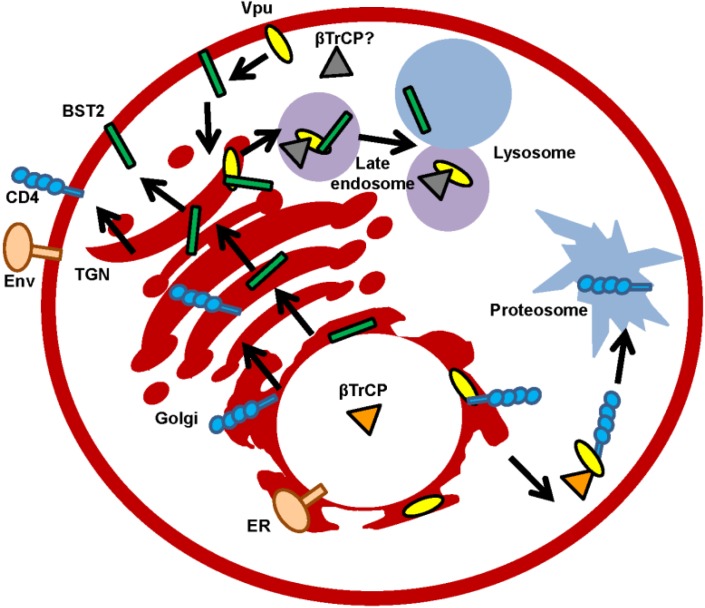
Subcellular localizations of Vpu protein. Vpu modifies the protein transport of newly synthesized proteins. Degradation through the lysosome pathway (illustrated with Bone Marrow Stromal Cell Antigen 2 (BST2)) or the proteasome pathway (illustrated with CD4) are included. β-transducing-repeat-containing *protein* (βTrCP) or unknown cellular cofactor and also the two possible locations (trans-Golgi network (TGN) and plasma membrane (PM)) where BST2 and Vpu converge and traffic are depicted.

Vpu is non-packed into virions though co-localizes with Env within the TGN where the viroporin primarily resides. Other localizations where Vpu has been found are the endosomal system, plasma membrane (PM), and the ER. Notably, Vpu does not oligomerize in the ER, but does so in the Golgi region or in post-Golgi vesicles [46]. Thus, it is possible that different functions for monomeric and oligomeric forms of Vpu exist, which are dependent on the membrane compartment (Figure 2).

Together with the Nef protein, Vpu modifies the subcellular distribution of membrane proteins, endowing infected cells with optimal assembly and budding at membrane microdomains [47].

The downregulation of several membrane proteins by Vpu, including cell surface receptors, viral glycoproteins, and antiviral factors, has been reported [48] (Table 1). Among them, CD4 and BST2 stand out because of their special biological significance for virus transmission.

**Table 1:** Proteins modulated by Vpu. Cellular and viral proteins respond to the presence of intracellular Vpu by altering their trafficking or intracellular concentration. The table includes membrane proteins exposed to the cell surface (blue), proteins involved in protein trafficking or virus release (purple), K+ channel (pink) and virus-encoded proteins (green).

Protein	Modification	References
BST2/tetherin/CD317	downregulation/localization	[24]
CCR7	downregulation	[49]
CD1d	downregulation	[50,51]
CD4	downregulation	[11]
CD40	upregulation	[52]
CD81	downregulation	[53]
CD155/PVR	downregulation	[54,55]
CDL62	localization	[56]
MHC-I	downregulation	[57]
NTB-A	localization?	[58,59]
AP-1	localization	[60]
βTrCP	localization	[61]
TSPANs (25, 26, 28 and 30)	downregulation	[62]
TASK-1	downregulation	[40]
Env (HIV-1)	localization	[7,63]
Gag (HIV-1)	localization	[63,64]
Glycoprotein (SV)	processing/downregulation	[65]

It is now well established that Vpu targets CD4 and BST2 proteins for degradation in a β-transducing-repeat-containing protein (βTrCP)-dependent manner [66,67]. Cells expressing Vpu redistribute βTrCP from the nucleus to the cytoplasm [61]. The signal for the recognition of Vpu by βTrCP is the phosphorylation of one or two serine residues present in a conserved motif, DSGXXS. Other elements in the cytoplasm regulate Vpu-induced downregulation of BST2. A putative trafficking motif, EXXXLV, in the second α-helix of the cytoplasmic domain of Vpu directs Vpu-bound membrane protein into the endosomal degradation pathway. In the absence of this motif, Vpu/BST2 complexes are incorporated into nascent virions, and virus release from CD4+ T cells becomes impaired [68]. The proposed general model to explain the Vpu-induced degradation of membrane proteins requires the interaction of Vpu and the targeted protein via their transmembrane domains. Subsequently, the recruitment of the cofactor βTrCP allows the formation of a ternary complex that targets substrates, such as CD4 or BST2, to degradation via the ubiquitin proteasome pathway [28,69]. Alternatively, in the case of BST2, another model has been proposed whereby Vpu induces the endosome-lysosome pathway and endosomal sorting complexes required for transport (ESCRT)-mediated degradation of BST2 [70,71,72]. Interestingly, recent NMR studies have shown that the cytoplasmic domain of Vpu interacts with lipids at the plasma membrane via the W76 residue, and this interaction specifically displaces BST2 from viral assembly sites [32]. Thus, as discussed below, Vpu variants may show impaired ability for BST2 degradation while preserving the enhancement of virus release [73].

### Vpu Adjusts the Protein Composition and Functioning of Cellular Membranes

In addition to the degradation of membrane proteins discussed above, Vpu induces the diversion of membrane proteins from their intrinsic trafficking pathways to alternative compartments away from degradation. This is the case for Env and Group-specific antigen (Gag) viral proteins which, in the absence of Vpu, accumulate in endosomes, but in the presence of Vpu, traffic to the plasma membrane for virus assembly and budding [63,64,74]. The role of Vpu in the subcellular transport of both virion components is cell-type dependent and occurs even when virion assembly takes place in late endosomes and virus release through the exosomal pathway [75,76,77]. Early studies suggested that the ion channel activity of Vpu may affect the trafficking of membrane proteins across internal cellular membranes [21]. Dissipation of ionic gradients within the endosomal system by Vpu may promote the recruitment of the ESCRT machinery required for budding [63,78]. This model was corroborated by the observation that membrane depolarization correlates with Vpu-mediated virus egress by budding [39]. The role of Vpu viroporin in the modulation of virus dissemination through direct cell-to-cell contacts at the virological synapse remains to be clarified [79,80].

The difficulty to elucidate the structure and conformation of membrane proteins in their native membrane environment limits the characterization of Vpu-formed channel/pore structures [81,82]. NMR and molecular dynamics simulation studies proposed that oligomerized Vpu forms ion channels with weak cation selectivity [35,83,84]. Additionally, functional approaches using artificial membranes or cellular models revealed that full-length Vpu protein permeabilizes membranes to cations, amino acids and also charged molecules such as hygromycin B and neurobiotin [21,36,85]. Thus, there is an ongoing debate about whether Vpu should be considered as a strict ion channel or rather as a pore [86,87].

## 4. Conservation and Heterogeneity of the Vpu Protein among Human and Non-Human Primate Isolates

Primate lentiviruses have acquired “accessory” genes that allow continuous and efficient viral replication despite apparently strong innate and acquired immune responses [88]. The genome of the human lentivirus HIV-1 and also some of the simian lentivirus, such as simian immunodeficiency virus (SIV)cpz, SIVgsn, SIVmon, SIVden, SIVmus and SIVgor, include the accessory gene *vpu* [89]. The genome of human HIV-2 lacks the *vpu* gene, but encodes an envelope glycoprotein that displays Vpu-like activity [90].

### 4.1. Conservation of Vpu Functions in Natural Isolates

The recent availability of Vpu sequences has provided new insights into lentivirus evolution and the contribution of Vpu to viral pathogenesis. Comparative studies of natural isolates showed that Vpu is one of the most highly variable proteins in the HIV-1 proteome [91]. The three HIV-1 phylogenetic groups, M (main), O (outlier), and N (non-M, non-O), can be discriminated by the ability of their corresponding Vpu proteins to downregulate CD4 and BST2 proteins [92] (Table 2). Vpu protein from the rare N strains lacks anti-CD4 activity, whereas Vpu proteins from the non-pandemic O group are poor BST2 antagonists [93]. Additionally, Vpu proteins from the global pandemic M strains are fully functional for antagonistic CD4 and BST2 activities. Moreover, Vpu protein from the low spread O group is retained in the ER rather than in the TGN, as occurs in the M group. In agreement with these observations, group O isolates use the Nef protein to antagonize BST2 [94]. Conceivably, viruses have evolved to counteract this antiviral factor [95].

**Table 2:** Host-adaptation activities of Vpus from each HIV-1 group. The putative P group is extremely rare and is thought to originate from a virus transmission from gorillas [96]. Viruses from M, N and O groups originate from SIV strains found in chimpanzees [97].

Group	CD4 Downregulation	BST2 Downregulation	Localization of Vpu	References
P?	no	no	?	[98,99]
N	no	poor	TGN	[100]
O	yes	Nef-mediated	ER	[93,99]
M	yes	yes	TGN	[100]

Nevertheless, the trafficking motif, EXXXLV, is well conserved in Vpu from A, B, D, G, and H subtypes of the M group and also from the O group. It is not clear, however, whether this Vpu motif acts in the TGN or in the plasma membrane [68,101]. The above-mentioned W76 residue in the Vpu cytoplasmic domain is well conserved in Vpu proteins from B, D, G, and J subtypes of the M group; however, a W76G polymorphism could be detected during the course of infection and also in most F subtype isolates [32,102]. Vpu proteins with a W76G substitution were impaired in their ability to enhance virion release despite preserved activity in the downregulation of BST2 protein from the cell surface.

### 4.2. Sequence Requirement of Vpu Functions

The majority of functional and computer simulation studies on Vpu have been performed with Vpu proteins from the M group, specifically from the subtype B such as HXB2/HXB3 isolates. Site-directed mutagenesis studies with Vpu_NL4-3_ from a laboratory-adapted virus showed that mutation S52N, S56N at the βTrCP binding site renders Vpu unable to downregulate BST2 but partially capable of enhancing virion release [29,70]. Other studies suggested that S23 was essential for Vpu ion channel activity, although most isolates from group M carry the S23T substitution [86,89]. The S23A mutation impairs Vpu ion channel activity in patch-clamp experiments in 293T cells but does not affect its interaction with BST2 [55,103]. Similarly, mutations at conserved A10, A14 or A18 residues of the Vpu transmembrane domain impaired particle release and downregulation of the BST2 surface expression activities, but not downregulation of the CD4 surface expression or ion channel activity [103]. Furthermore, recent data have shown that over the course of infection, the Vpu amino acid sequence can be highly variable within an individual [104]. Despite the high variation of Vpu protein within one infected individual, the downregulation of CD4 and BST2 and also the enhancement of virion release are strictly maintained throughout HIV-1 infection [104]. Evidently, these novel data confirm the relevance of Vpu not only for virus spread but also for its *in vivo* persistence in infected individuals. The conservation of other Vpu activities, such as channel/pore forming, awaits further investigation. Presumably, the comparison of sequences from transmitted/founder and chronic viruses will reveal new determinants for each Vpu function [102].

## 5. Vpu Viroporin as a Therapeutic Target

The contribution of Vpu to virus transmission and also its persistence along the disease progression highlight the considerable therapeutic potential of this viroporin [104]. To date, the majority of efforts to develop specific anti-Vpu drugs have concentrated on the ability of viroporins to disturb ionic currents in membranes. This strategy has led to the search for inhibitors of other viroporins [105]. The first approaches to search for Vpu inhibitors utilized the measurement of ionic currents in artificial planar lipid bilayers [106], a methodology that led to the discovery of 5-(N,N-Hexamethylene) amiloride (HMA) as the first Vpu inhibitor [107]. Further evaluation of HMA in cellular models, unfortunately, demonstrated HMA to have low effectiveness due to its low selectivity index. Additional studies with amiloride analogues resulted in the discovery of a second inhibitor of Vpu ion channel activity, BIT225 [108]. The synthesized drug also inhibits the ion channel activity of the hepatitis C virus (HCV) viroporin, p7 [109]. This new compound demonstrated a superior selectivity index and has entered into phase two clinical trials. Recent data released from the BIT225 licensing company suggest its efficacy in HIV/HCV coinfected patients when used in combination with HCV drugs.

Evidence for functional complementation between Vpu and other viroporins have provided an alternative infection context to assess Vpu activity [65,110]. Thus, a single amino acid substitution in the Vpu transmembrane domain of a chimeric SIV/HIV virus (A19H) resulted in virus sensitization to rimantadine [111]. Consequently, the transmembrane domain of Vpu was proposed as an antiviral target. Further NMR structural analysis showed how this local change in the primary sequence affects secondary and tertiary structures of Vpu transmembrane peptide in lipid bilayers and alters drug binding to the viroporin [112]. Other recent studies have proposed alternative cellular models that are suitable for screening of inhibitors of full-length Vpu. Notably, the K+ dependent growth stimulation caused by Vpu protein in *S. cerevisiae* and also in *Escherichia coli* provides efficient cellular platforms to develop high–throughput screening assays [41,113]. Finally, *Xenopus* oocytes have also been used for drug testing for anti-Vpu activity by voltage clamp assays [114]. Clearly, inhibitors preselected with these phenotypic assays should be further validated in viral infections with representative pandemic strains.

## 6. Conclusions and Perspectives

Vpu viroporin is prominently involved in the spread of HIV-1 from infected cells. The precise mechanisms underlying this activity remain to be characterized. Nonetheless, the increase in number of available laboratory models to analyze Vpu separated from other cytotoxic viral proteins and the scope of sequences from natural isolates are providing new insights into the Vpu mode of action. Cell-type dependency for most of the Vpu functions may explain its apparent redundant and even opposite modes of functioning. Several lines of evidence indicate that Vpu antagonizes host restriction factors that interfere with virus dissemination. Vpu also modifies the composition and functioning of cellular membranes, which become adjusted to viral requirements at late stages of the virus life cycle. The overall architecture of Vpu seems to be essential for its functionality, more so than the specific amino acid sequence. Indeed, the variable supramolecular organization in subcellular compartments and also the turnover of the viroporin may modulate the contribution of Vpu’s pleiotropic functions to the enhancement of virus spread. Further studies should clarify the differential mode of action of Vpu in cell-free and cell-cell virus transmissions and in plasma membrane or multivesicular virion assemblies. So far, there is no specific anti-Vpu drug that has passed clinical trials further than phase two. Nevertheless, available cell-based phenotypic screening assays are ready to search for anti-Vpu drugs.
